# Augmenting Bone Formation by Implanting Dedifferentiated Fat Cell-Loaded Cotton-like Graft Materials in a Rat Bone Defect Model

**DOI:** 10.3390/bioengineering12121364

**Published:** 2025-12-16

**Authors:** Jin Inoue, Tomohiko Kazama, Takahisa Okubo, Daisuke Akita, Yoshinori Arai, Yoshiyuki Hagiwara, Koichiro Kano, Masaki Honda, Taro Matsumoto

**Affiliations:** 1 Division of Applied Oral Sciences, Nihon University Graduate School of Dentistry, Tokyo 101-8310, Japan; deji22005@g.nihon-u.ac.jp; 2 Department of Partial Denture Prosthodontics, Nihon University School of Dentistry, Tokyo 101-8310, Japan; okubotakahisa@gmail.com (T.O.); hagiwara.yoshiyuki@nihon-u.ac.jp (Y.H.); 3 Department of Functional Morphology, Division of Cell Regeneration and Transplantation, Nihon University School of Medicine, Tokyo 173-8610, Japan; kazama.tomohiko@nihon-u.ac.jp (T.K.); matsumoto.taro@nihon-u.ac.jp (T.M.); 4 Department of Oral and Maxillofacial Radiology, Nihon University School of Dentistry, Tokyo 101-8310, Japan; arai.yoshinori@nihon-u.ac.jp; 5 Laboratory of Cell and Tissue Biology, College of Bioresource Science, Nihon University, Fujisawa 252-0880, Japan; kano.kouichirou@nihon-u.ac.jp; 6 Department of Oral Anatomy, Aichi Gakuin University School of Dentistry, Nagoya 464-8650, Japan; honda-m@dpc.agu.ac.jp

**Keywords:** beta-tricalcium phosphate, bone augmentation, dedifferentiated fat cells, poly (L-lactide-co-glycolide)

## Abstract

Bone graft materials frequently employed in dental implant placement procedures, including hydroxyapatite and β-tricalcium phosphate (β-TCP), are typically granular in form, which complicates their manipulation and contributes to extended treatment durations. A tissue engineering approach utilizing readily manageable biomaterials in conjunction with mesenchymal stem cells (MSCs) represents the most promising approach in dentistry. This study assessed the bone-augmenting capacity at bone defect sites in inbred rats by seeding dedifferentiated fat (DFAT) cells onto a cotton-like bone graft scaffold composed of β-TCP and poly(L-lactic-co- glycolide) (PLGA), which was subsequently wrapped around titanium. As a control, cotton-like bone graft material without cells was used and wrapped around and transplanted. Four weeks post-implantation, computed tomography (CT) images of the DFAT group revealed a 1.25-fold enhancement in hard tissue formation compared to the control group. Histological analysis revealed compact structure in a dark red color surrounding the cotton-like bone graft material was observed on the titanium surface of DFAT group. Histomorphometric analysis revealed that the amount of hard tissue generated in the DFAT group was approximately 2.5 times higher than that observed in the control group. Moreover, this mineralized tissue demonstrated properties analogous to those observed in cortical bone. Collectively, these findings indicate that the composite of DFAT cells and cotton-like bone graft material holds potential for bone augmentation applications and represents a promising approach for regenerative therapies within the orofacial region.

## 1. Introduction

The dimensions of the alveolar bone, specifically its height and width, that experience atrophic alterations following tooth loss, are critically important for the stability of removable dentures and the successful placement of dental implants. In dental implant restorations, the alveolar bone serves as the direct support for the implant fixture via a functional integration process known as osseointegration. Contemporary dental implants are predominantly inserted into the jawbone; therefore, the restoration of atrophied jawbone continues to present a significant clinical challenge [1,2]. To effectively overcome these challenges, the implementation of sophisticated methodologies is essential, given the critical roles of angiogenesis, cellular proliferation, and osteogenesis [3]. Autogenous bone grafts, typically obtained from either extraoral or intraoral locations, are widely regarded as the gold standard for reconstructing maxillofacial bony defects due to their osteogenic, osteoconductive, and osteoinductive properties [4,5]; however, this procedure presents considerable clinical challenges, including wound infection, hematoma formation, pain, and gait disturbances [6,7]. Consequently, the guided bone regeneration (GBR) technique, which combines various bone substitute materials characterized by slow absorption rates and the ability to maintain the necessary space with barrier membranes that inhibit the infiltration of unwanted soft tissues into osseous defects, has become increasingly favored in clinical settings for mobilizing host-derived osteoblasts and their precursor cells to achieve alveolar ridge augmentation [8,9,10,11]. However, because the treatment period is prolonged, widespread clinical adoption remains limited [12].

Recent developments in regenerative medicine have introduced tissue engineering strategies that utilize porous scaffolds in conjunction with membranes to preserve spatial integrity. These approaches, when combined with mesenchymal stem cell (MSCs)-based techniques, offer potential solutions to address several of the existing therapeutic challenges [13,14]. MSCs, characterized by their capacity for proliferative and multipotent differentiation, exert paracrine effects, provide trophic support, and modulate immune responses [15]. These properties render them highly promising candidates for applications in regenerative medicine, particularly in facilitating bone regeneration or augmentation [12]. To facilitate cell-based bone regeneration or augmentation within the oral cavity, an adequate quantity of MSCs is essential. Consequently, MSCs must be procured from cell sources around the oral region, subsequently cultured and expanded ex vivo, and finally transplanted to the designated site [16].

MSCs can be derived from multiple sources; however, adipose tissue is regarded as a particularly promising source for regenerative medicine due to its accessibility from both the abdominal and midfacial regions. Dedifferentiated fat cells, referred to as DFAT cells and derived solely from floating mature adipocytes, represent a stem cell technology based on adipose tissue. These cells form a homogeneous population characterized by a high capacity for proliferation and multipotent differentiation potential [17,18,19]. In our previous study, we established that dedifferentiated fat (DFAT) cells possess a superior potential for osteoblastic differentiation compared to adipose-derived stromal cells (ASCs). Moreover, when DFAT cells were seeded on poly(lactic-co-glycolic acid) (PLGA) scaffolds and implanted into periodontal fenestration defect models in inbred rats, successful regeneration of periodontal tissue was achieved [20]. Adipose tissue, the cellular origin of DFAT cells, can be obtained via intraoral approaches, and the isolation of DFAT cells involves relatively straightforward procedures. Given that DFAT cells comprise a highly purified cell population, they represent an optimal cell source for applications within the field of dentistry. Therefore, while establishing protocols for cell manipulation, cell processing, and transportation is essential, there is significant potential to shorten the treatment period and improve bone augmentation compared to using bone graft materials.

PLGA is among the most widely utilized synthetic biodegradable polymers, distinguished by its biocompatibility, biodegradability, flexibility, and minimal adverse effects. These attributes collectively render PLGA highly advantageous for various biomedical applications [21]. PLGA has been reported to be effective in a wide range of dental applications, including the development of screws for bone fixation [22,23,24], treatment of periodontal pathogens [25], fabrication of oral mucosa [26], and indirect pulp capping procedures [27,28]. PLGA materials are available in multiple configurations, including membranes, scaffolds, films, nanoparticles, and microparticles, and they demonstrate excellent biocompatibility in dental disciplines focused on the regeneration of damaged tissues, including root canal treatment, periodontal treatment, implantology, and stem cell-based interventions [29]. Recently, a novel biodegradable bone graft material with a cotton-like morphology has been engineered by incorporating osteoconductive β-tricalcium phosphate (β-TCP) particles into the PLGA matrix. This composite was subsequently processed into fibrous structures with diameters ranging from 40 to 320 μm through the technique of electrospinning [30]. This material exhibits ease of manipulation and is an appropriate grafting substance for the reconstruction of extensive bone cavities characterized by intricate defects or constricted apertures [31]. Recent studies have reported that cotton-like β-TCP/PLGA bone graft material has the ability to promote new bone formation in a rabbit maxillary sinus floor elevation model [32]. To date, no investigations have been conducted to evaluate this cotton-like bone graft material in conjunction with cellular components. Consequently, the present study involved seeding DFAT cells onto the cotton-like bone graft material, followed by transplantation into a rat femoral bone defect model, with the objective of assessing its osteogenic potential. Subsequently, the null hypothesis was that there is no difference in the effects of implanting cotton-like bone graft material loaded with DFAT cells. In contrast, the alternative hypothesis was that the implantation of cotton-like bone graft material containing DFAT cells exerts a significant effect. To evaluate this, the quantity of hard tissue formation surrounding titanium was measured using micro–computed tomography (CT) in conjunction with bone analysis software. Furthermore, the efficacy of the DFAT cell-loaded graft material was assessed through histological and histomorphometric analyses. A significance level of 5% was adopted for statistical testing. Should the results indicate a statistically significant difference, the null hypothesis will be rejected.

## 2. Materials and Methods

### 2.1. Ethical Considerations

All experimental procedures were approved by the Animal Research and Care Committee of Nihon University School of Dentistry (Approval Nos.: AP22DEN021-3 and AP22MED030-2) and were conducted following institutional regulations. Nine-week-old male F344 rats (body weight 190 ± 10 g) were purchased from CLEA Japan, Inc. (Tokyo, Japan).

### 2.2. DFAT Cell Preparation

**Figure 1:**
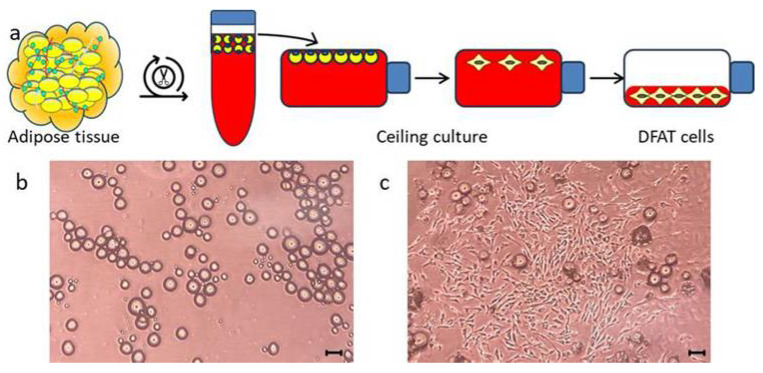
Preparation of DFAT cells and microscopy images of the flask surface. (**a**) By harnessing the ceiling culture method, floating mature adipocytes, which have been isolated via enzymatic digestion, are cultivated in a medium-filled flask, resulting in the generation of proliferative fibroblast-like cells. (**b**) Day 0: Microscopic image depicting the upper surface of the flask immediately following the initiation of ceiling culture. (**c**) Day 7: Microscopic image captured during ceiling culture, depicting the inverted top surface of the flask. Fibroblast-like cells are observed to be forming colonies originating from isolated mature adipocytes.

Six male F344 rats were used as previously described [20]. Approximately 1 g of subcutaneous fat was digested with 0.1% collagenase (C6885; Sigma-Aldrich, St. Louis, MO, USA) at 37 °C for 30 min. Floating adipocytes were isolated, washed, and cultured in Dulbecco’s modified Eagle’s medium (DMEM; Gibco, Waltham, MA, USA) supplemented with 20% fetal bovine serum (FBS; Nichirei Bioscience Inc., Tokyo, Japan Lot 22D223) at 37 °C, 5% CO_2_. After 1 week of ceiling culture, flasks were inverted to allow cell attachment to the bottom surface. First-passage DFAT cells were used for all experiments (Figure 1).

### 2.3. Fabrication of DFAT Complexes

**Figure 2:**
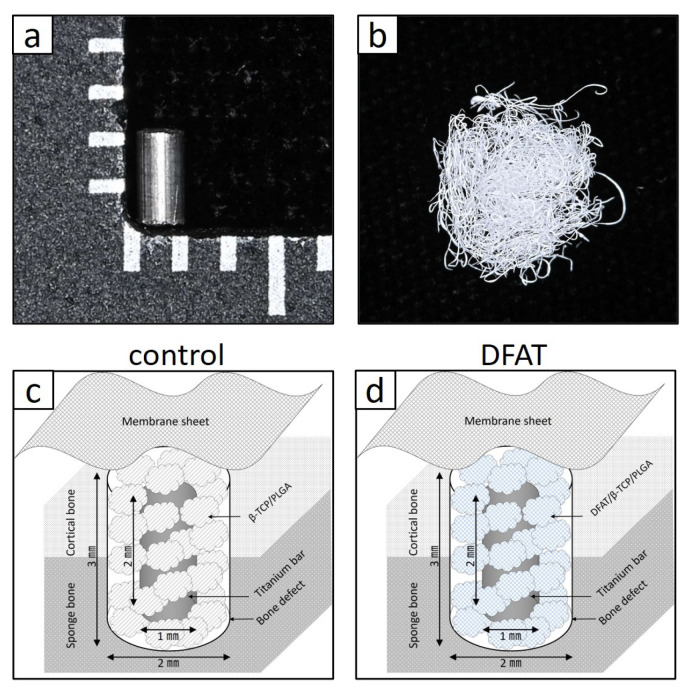
Materials and schematic diagrams used for the in vivo experiment. (**a**) Type IV pure titanium in a cylindrical shape with a diameter of 1 mm and a height of 2 mm the machine surface (**b**) Cotton-like bone graft materials based on β-tricalcium phosphate (β-TCP)/poly(L-lactide-co-glycolide) (PLGA). (**c**) Schematic diagram of the control group; (**d**) Schematic diagram of the experimental group. In both groups, titanium was wrapped in moist cotton-like bone graft material, inserted into an artificially created bone defect and covered with a membrane sheet. In (**d**), DFAT cells were seeded onto the cotton-like grafts before insertion.

The DFAT complex consisted of a titanium bar, cotton-like bone graft materials, and DFAT cells. The titanium bar (YUMOTO ELECTRIC Inc., Osaka, Japan) was a cylindrical pure titanium (Type IV) specimen measuring 1 mm in diameter and 2 mm in height, with a machined surface (Figure 2a). First, we purchased 200 mg of cotton-like bone graft material (ReBOSSIS-J^®^; ORTHOReBIRTH, Kanagawa, Japan), which was manufactured by dissolving about 70 wt% of 1.0–5.0 μm bone-conductive β-TCP particles in 30 wt% PLGA, then processing it into fibrous forms with diameters of 40–320 μm using electrospinning through a large-diameter nozzle [30] (Figure 2b).

For the control group, 1.5 mg of cotton-like bone graft materials were cut into pieces approximately 3 mm square and wrapped around the titanium bar without the addition of cells. The complex was immersed in DMEM with 20% FBS and incubated under the same conditions (37 °C, 5% CO_2_) for approximately 6 h before implantation (Figure 2c).

Concurrently, DFAT cells (1.0 × 10^6^) were seeded into 1.5 mg of cotton-like bone graft materials [33], which were subsequently wrapped around the titanium bar following the same procedure employed for the control group. The assembled complex was immersed in DMEM supplemented with 20% FBS and incubated at 37 °C, 5% CO_2_ for approximately 6 h prior to implantation (DFAT group; Figure 2d).

### 2.4. Implantation Procedure

**Figure 3:**
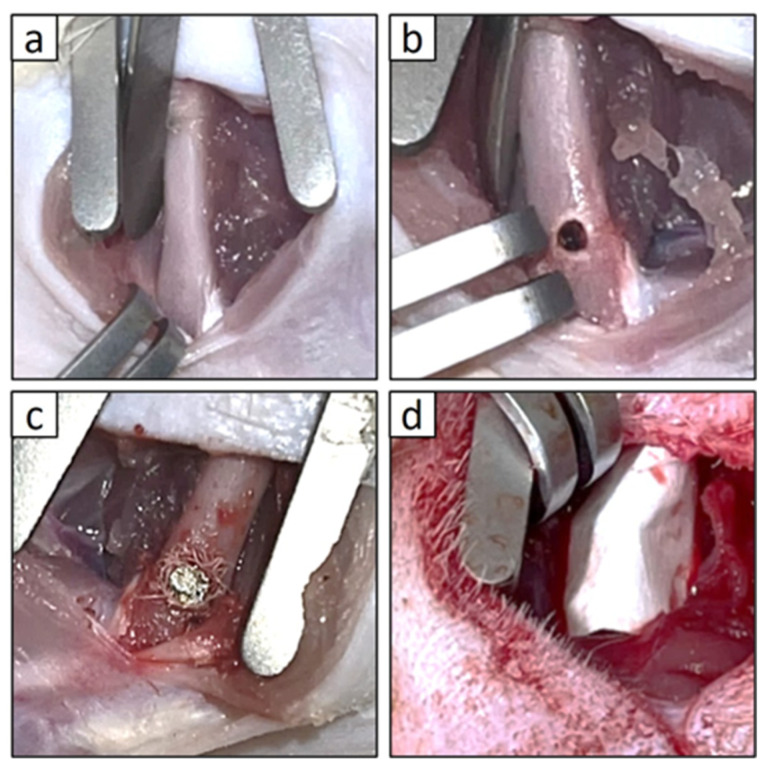
Magnified images of the in vivo implantation experiment. (**a**) Exposed femur of a depilated rat. (**b**) A bone defect approximately 2 mm in diameter and 3 mm in depth, created 10 mm away from the distal end of the femur. (**c**) Implantation of titanium wrapped in moist cotton-like bone graft material into an artificially created bone defect. (**d**) The entire surgical site encased with a membrane sheet.

Twelve healthy male F344 rats were used for the in vivo implantation study. A femoral implant bed model was prepared under inhalation anesthesia with isoflurane (VTRS; Viatris Inc., Canonsburg, PA, USA). The hindlimbs were shaved and disinfected with 10% povidone–iodine, followed by a skin incision and muscle dissection to expose the distal femur (Figure 3a).

The implantation site was located 10 mm proximal to the distal femoral condyle. A water-cooled 1.0 mm round bur was used for initial drilling, followed by reaming with sterilized dental reamers (#90 and #100; MANI, Inc., Utsunomiya, Tochigi, Japan) to create a cylindrical cavity measuring 2 mm in diameter and 3 mm in depth (Figure 3b). The preprepared titanium complex was carefully implanted into the trabecular bone, avoiding any contact with the cortical bone, and subsequently covered with a collagen membrane (Colla Tape; Zimmer Dental Inc., Carlsbad, CA, USA) to prevent displacement, and sutures were placed (Figure 3c,d) [34].

The right femur of each rat was used for analysis, and animals were randomly assigned to two groups: DFAT group—titanium bars with cotton-like bone graft materials seeded with DFAT cells; Control group—titanium bar with cotton-like bone graft materials without DFAT cells. A sample size of six animals per group was established to adequately capture biological variability, generate reliable preliminary data, and adhere to the ethical principles of animal research, specifically the 3Rs (Replacement, Reduction, and Refinement). Post-transplantation, all animals received pain management through the administration of an oral dose of carprofen at a concentration of 1 mg/kg in accordance with the animal experiment protocol.

### 2.5. Micro-Computed Tomography Scanning and Evaluation

As previously described, hard tissue formation was assessed using a micro–CT (R_mCT; Rigaku Corporation, Tokyo, Japan) [20]. The exposure parameters were as follows: tube voltage, 90 kV; tube current, 100 μA; magnification, 6.7×; exposure time, 17 s; and isotropic voxel size, 30 μm.

Scans were performed immediately after implantation and at 4 weeks postoperatively. Three-dimensional reconstruction was conducted using i-View software version 1.74 (J. Morita Co., Kyoto, Japan). The bone volume (BV) was measured using a bone-analysis software (3by4viewer 2024), with the region of interest (ROI) set at 1.8 mm by 3.6 mm to encompass the graft site adjacent to the titanium. The increase in mineralized tissue was calculated by subtracting the immediate postoperative BV from the BV at 4 weeks.

### 2.6. Histological and Histomorphometric Evaluations

To minimize bias, all analyses were performed independently by two investigators who were blinded to the group assignments. Specimens were fixed in 10% neutral-buffered formalin for 24 h, dehydrated in ethanol for 10 days, defatted with xylene for 3 days, substituted with acetone for 3 days, and embedded in resin. Ground sections (70 μm) were stained with hematoxylin–eosin (HE) and Villanueva–Goldner (VG). For HE staining, the deresinated tissue sections were initially stained with iron hematoxylin for 20 min, followed by treatment with a 1% hydrochloric acid–ethanol solution. Subsequently, the sections were stained with eosin solution and rinsed using 99.5% ethanol. Newly formed hard tissue along the lateral surface of the titanium rod was evaluated on H&E-stained sections using a light microscope (BZ-X800; KEYENCE, Osaka, Japan) at ×20 magnification. The bone-to-implant contact (BIC) ratio within 50 μm from the titanium surface on both lateral aspects of the rod was quantified using image analysis software [35]. The BIC was calculated as the area of newly formed hard tissue within 50 μm from the titanium surface on both lateral sides divided by the corresponding titanium surface area within the same 50 μm region. During the specimen preparation phase, one sample was lost during the sectioning process; consequently, five specimens were subjected to evaluation. For VG staining, the deresinated sections underwent the following procedure: staining with Cole’s hematoxylin for 10 min, treatment with a 1% hydrochloric acid–ethanol solution, staining with ponceau fuchsin for 2 min, treatment with 1% acetic acid, and staining with a phosphotungstic acid–phosphomolybdic acid solution for 5 min. Finally, the sections were stained with Naphthol Green B solution (FUJIFILM Wako, Osaka, Japan) for 15 min and washed with ethanol solutions ranging from 70% to 99.5% [36]. The VG staining analysis revealed that calcified hard tissue was indicated by a green coloration, whereas immature hard tissue was represented by a purple hue.

### 2.7. Statistical Analysis

Data were expressed as mean ± standard deviation. Normality was not assumed due to small sample size; therefore, a non-parametric Mann–Whitney U test was used.

## 3. Results

### 3.1. Micro-CT Analysis

**Figure 4:**
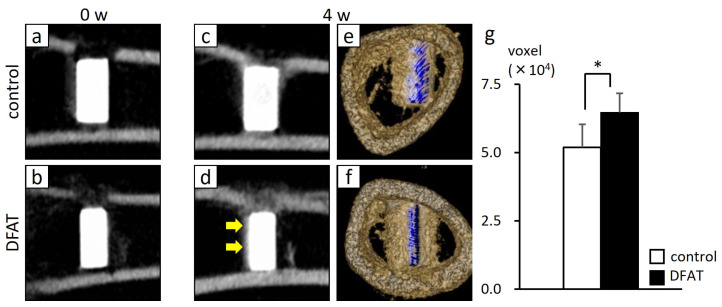
Computed tomography (CT) and reconstructed images at 0 weeks and 4 weeks post-implantation, and quantitative analysis of newly formed hard tissue formation. (**a**,**b**) CT images of the control group and the dedifferentiated fat cells (DFAT) group immediately after surgery. (**c**,**d**) CT images of the control group and DFAT group at 4 weeks post-surgery. The yellow arrows indicate areas of increased opacity. (**e**,**f**) Reconstructed images of the control group and the DFAT group. The blue areas indicate the titanium bar, and the brown areas indicate hard tissue. (**g**) Quantification of newly formed hard tissue surrounding the titanium bar using bone volume measurement software. The DFAT group demonstrated approximately 1.3 times greater hard tissue regeneration compared to that in the control group. Mean ± standard deviation values were 51,393.16 ± 8318.41 for the control group and 64,611.17 ± 7039.23 for the DFAT group (*n* = 6, * *p* < 0.05).

A standardized femoral defect was surgically created, and either the DFAT or the control complex was implanted. To evaluate hard tissue formation around the titanium bar, sagittal CT images at 0 and 4 weeks, as well as reconstructed CT images, were obtained. Micro–CT imaging revealed an increase in radiopacity in both the control and DFAT groups. At 4 weeks, both groups showed restoration of the superior cortical bone continuity at the defect site. In the DFAT group, however, the increase in radiopacity around the titanium bar was more pronounced than that in the control group. Three-dimensional reconstructed images demonstrated that newly formed hard tissue was more extensively distributed around the titanium bar in the DFAT group. Quantitative analysis of the hard tissue surrounding the titanium bar, performed using bone volume measurement software, revealed that the volume of newly formed hard tissue was significantly higher in the DFAT group compared to the control group (Figure 4) (* *p* < 0.05)

### 3.2. Histological Analysis

**Figure 5:**
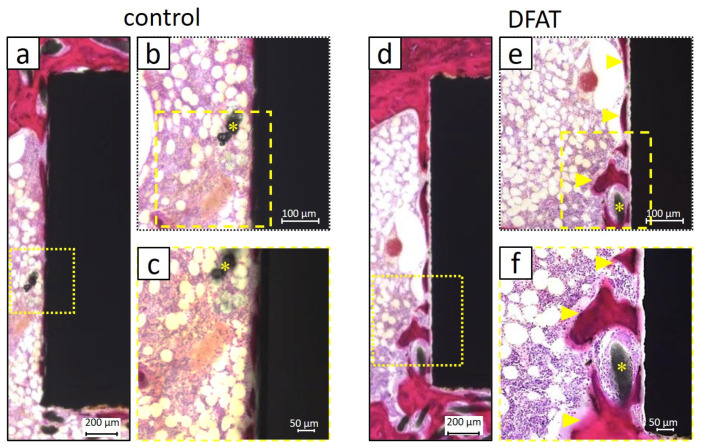
Representative hematoxylin and eosin (H&E)-stained histological sections at 4 weeks post-surgery, in which bone is presented as a compact structure in a dark red color. (**a**–**c**) control group and (**d**–**f**) DFAT group. The asterisks indicate unabsorbed cotton-like bone graft material, the arrowheads indicate the newly formed hard tissue. In the control group, only the implanted titanium bar and unabsorbed cotton-like bone graft material are observed. In the DFAT group, newly formed hard tissue without interposition of soft tissue could be detected around the implanted titanium bar.

Hematoxylin–eosin (HE) staining demonstrated the existence of mineralized tissue encasing the titanium bar in both the control and DFAT groups (Figure 5a,d). In the control group, graft material residues were observed on the implanted titanium surface (Figure 5b,c). Conversely, in the DFAT group, eosin-positive hard tissue formation was intermittently observed on the titanium surface. Moreover, graft material remnants encased by newly formed hard tissue were also observed (Figure 5e,f).

### 3.3. Histomorphometric Analysis

**Figure 6:**
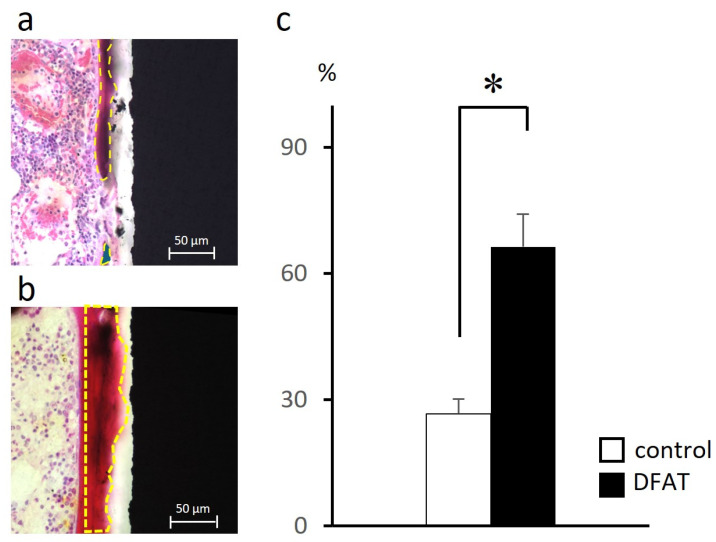
Representative HE-stained image of newly formed mineralized tissue observed on the titanium surface and bone-to-implant contact (BIC) value calculation. (**a**) The control group; (**b**) The DFAT group. In both groups, the region of interest (ROI) was defined as the area within 50 µm from the titanium surface, as indicated by the yellow dashed outline. (**c**) BIC values calculated using image analysis software. A significant difference was observed between the control and DFAT groups. Mean ± SD values were 26.6 ± 3.4 for the control group and 66.3 ± 7.8 for the DFAT group (*n* = 5, * *p* < 0.05).

HE–stained sections revealed differences in the amount of hard tissue surrounding the titanium bar between the control and DFAT groups. Therefore, the measurement region was standardized, and quantitative analysis was performed to calculate the bone-to-implant contact (BIC) value (Figure 6a,b). The quantitative results are presented in the graph (Figure 6c). The amount of hard tissue formed around the titanium bar in the DFAT group was more than twice that observed in the control group.

### 3.4. Hard Tissue Maturation

**Figure 7:**
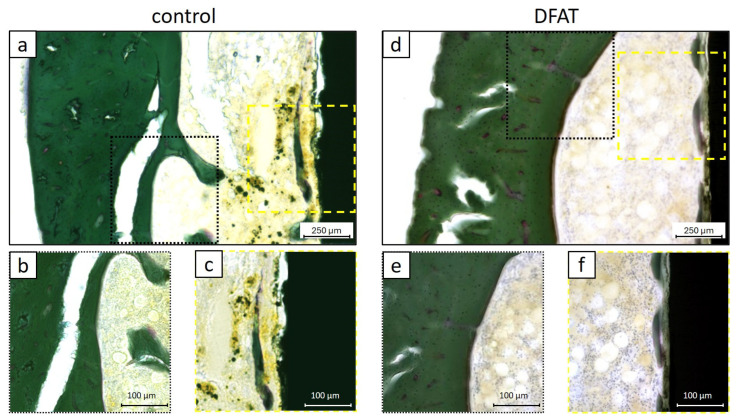
Representative histological sections stained with Villanueva–Goldner at 4 weeks post-surgery demonstrate the calcification of hard tissue formation on the titanium surface. (**a**,**d**) Low-magnification images of the grafted femoral region. (**b**,**e**) Enlarged images of the dotted area in (**a**) or (**d**), illustrating the cortical bone of the femur. (**c**,**f**) Enlarged images of the dashed area in (**a**) or (**d**), illustrate newly formed hard tissue on the implanted titanium surface. Both the control group and the DFAT group exhibited a level of calcification in the newly formed hard tissue comparable to that observed in cortical bone.

At 4 weeks post-implantation, Villanueva–Goldner staining demonstrated that both the control and DFAT groups presented newly formed hard tissue exhibiting a degree of calcification comparable to that of the cortical bone of the femur (Figure 7a,d).

In the control group, areas of hard tissue exhibiting a coloration similar to that of the femoral cortical bone were observed within the newly formed tissue surrounding the titanium bar (Figure 7b,c).

Similarly, in the DFAT group, mature hard tissue exhibiting staining characteristics comparable to the femoral cortical bone was observed on the titanium surface (Figure 7d–f).

## 4. Discussion

In this study, approximately 1 g of adipose tissue was isolated from the inguinal region of rats following a standardized protocol (Figure 1a). Mature adipocytes that migrated to the upper surface of the culture flask, buoyed by their inherent buoyancy within the culture medium, generated a population of fibroblast-like cells within approximately 1 week (Figure 1b,c). In this investigation, we limited the evaluation of the bone-augmenting ability of DFAT cell-loaded cotton-like bone graft material to in vivo conditions, specifically presumed to originate from the oral region. In a prior investigation, we demonstrated in vitro that DFAT cells derived from this specific strain of inbred rat exhibit a greater capacity for osteoblastic differentiation compared to ASCs obtained from the same individual [20]. Therefore, we did not examine the cellular characteristics of the DFAT cells; however, this cell population is derived exclusively from mature adipocytes and has been documented as a highly purified MSC-like population [19]. In the context of cell therapy involving animal-derived materials, such as FBS, established guidelines emphasize the necessity of confirming their safety and quality [37]. Accordingly, this study employed research-grade serum that had undergone rigorous quality assessments and was subjected to inactivation prior to its application in cell culture. Quality testing of the Panama-sourced FBS used in this study showed no detection of mycoplasma, tetracycline, cytopathic effect, hemadsorption, bovine virus diarrhea, bovine respiratory syncytial virus, bovine adenovirus, or other viruses. However, if the research progresses to translational studies in the future, it may be necessary to establish new protocols. In our previous study, we demonstrated that when DFAT cells seeded onto a PLGA scaffold and subsequently allogeneically transplanted into a rat model exhibiting periodontal tissue defects, a subset of these DFAT cells differentiated into the periodontal ligament, cementum, and alveolar bone tissues, thereby facilitating periodontal regeneration [20]. Nonetheless, no prior studies have investigated the role of DFAT cells in bone augmentation. Therefore, the present study employed cylindrical pure titanium as the experimental sample (Figure 2a). This pure titanium is produced via machining and is widely recognized for its superior biocompatibility, exceptional corrosion resistance, and high mechanical strength [38]. Furthermore, the development of a passive titanium oxide layer facilitates the intimate adhesion of physiological fluids, proteins, as well as both hard and soft tissues to the metal surface [39,40]. Building upon prior research demonstrating the efficacy of PLGA as a scaffold for DFAT cells, in this study we used β-TCP/PLGA, fabricated into a cotton-like structure via electrospinning, as a novel bone graft material (Figure 2b). This nanofiber cotton-like bone graft material exhibits superior operability and has demonstrated potential applications as a wound dressing and tissue engineering scaffold [41]; however, since in vivo studies are still limited, we aimed to assess its capacity to promote bone augmentation by applying it around a titanium bar (Figure 2c,d). For this purpose, a standardized quantity of cotton-like bone graft material was prepared in dimensions sufficient to envelop the titanium bar and subsequently utilized for transplantation. The cotton-like bone graft material, once moistened, was not pushed out from inside the bone defect that was larger than the diameter of the titanium. However, in future translational research, it may be imperative to precisely define the amount, size, and transplantation method of the bone graft material. In transplantation experiments, a standardized bone defect was surgically induced in the rat femur (Figure 3a,b). Within this experimental framework, bone augmentation was assessed using a titanium and DFAT complex under a controlled closed environment. Future studies should aim to investigate the bone augmentation capacity of this complex within the alveolar bone of the oral cavity, utilizing medium to large animal models. Subsequently, a titanium bar wrapped in cotton-like bone graft material was implanted into the defect site. In order to prevent direct contact between the created bone defect and the titanium bar, cotton-like bone graft material was applied both surrounding the titanium bar and on the floor of the socket (Figure 3c). To inhibit displacement of the titanium bar caused by the transplanted fibers within the bone defect, the transplantation site was ultimately covered with a membrane designed to restrict cellular migration (Figure 3d). A previous study employing membrane sheets similar to those utilized in the current study on collagen scaffolds seeded with DFAT cells demonstrated that the membrane inhibited epithelial down-growth and facilitated periodontal tissue regeneration in swine via autologous transplantation of DFAT cells [42,43]. Four weeks post-implantation, CT analysis of the DFAT group revealed the presence of hard tissue surrounding the titanium implant, demonstrating approximately a 1.25-fold increase in hard tissue regeneration relative to the control group (Figure 4a–g). These findings indicate that the cotton-like bone graft material made of β-TCP and PLGA was maintained around the titanium bars in both groups. Additionally, the DFAT group, exhibiting a larger volume of newly formed hard tissue than the control group it suggests that the DFAT cell-loaded cotton-like bone graft material enhances bone augmentation on the titanium surface. Another research group reported that when a cotton-like bone graft material was filled into the bone defect of the calcaneus in patients with rheumatoid arthritis, absorption of the material and new bone formation were observed on X-rays after four weeks, which contributed to the promotion of heel-walking training [44]. Accordingly, the transplantation period established in the present pilot study and the analysis using X-ray CT are considered to be useful indicators for evaluating bone augmentation.

In the HE-stained images at 4 weeks post-surgery, bone graft material remnants were observed around the implanted titanium in the control group (Figure 5a–c). Another research group using the same materials in a rabbit maxillary sinus lift model demonstrated that the transplanted β-TCP/PLGA remained up to 24 weeks post-surgery, and that the β-TCP gradually exposed by the hydrolysis of PLGA promoted the formation of hard tissue while maintaining the increased volume [32]. In the present study, the presence of residual bone graft material was also identified, corroborating findings reported in prior research. Conversely, within the DFAT group, intermittent hard tissue stained dark red was observed surrounding the titanium bar (Figure 5d,e). Additionally, hard tissue formation was evident encasing the transplanted bone graft material; however, no cellular presence was noted within this tissue (Figure 5f). Since the tissue specimens were sectioned with the titanium bar intact, it is likely that the section thickness influenced these observations. The absence of soft tissue between the titanium and the hard tissue suggests that the DFAT cell-loaded PLGA/β-TCP composite contributes to the process of osseointegration (Figure 6). The newly formed bone-like tissue confirmed around the titanium in both groups exhibited a level of calcification comparable to that of the cortical bone, indicating the efficacy of the bone graft material employed in this study for bone augmentation purposes (Figure 7). Previous studies have demonstrated that alterations to the surface characteristics of titanium influence MSCs [45,46], and that ASCs obtained from buccal fat pads exhibit enhanced cell viability following etching treatment of titanium disks [47]. Furthermore, Annunziata et al. concluded that nano- and micro-scale surface topographies contribute to the promotion of cellular differentiation, potentially influencing osseointegration [48]. As previously indicated, this study employed machined titanium as a preliminary investigation into DFAT cells and bone augmentation. Consequently, a passive titanium oxide layer was established on the titanium surface. Following implantation into the bone defect, it is postulated that proteins originating from blood and bone marrow, along with calcium (Ca^2+^) and phosphate (PO_4_^3−^) ions, adsorbed onto the surface, forming a monolayer [49]. It is further hypothesized that as the PLGA, utilized as a space maintainer, underwent hydrolysis, the β-TCP created a microenvironment favorable for osteoconduction [50,51]. Nonetheless, future investigations should consider evaluating the bone regeneration process with particular attention to the mechanical properties and additional attributes of this cotton-like bone graft material. The seeded DFAT cells secreted a substantial quantity of cytokines (growth factors), extracellular matrix components, and exosomes [52,53], which activated the adsorbed proteins and ions on the titanium surface, ultimately facilitating the formation of mineralized tissue. Nevertheless, given the relatively brief transplantation period in this study and a BIC ratio of approximately 60%, enhancing the experimental design—including modifications to transplantation parameters such as titanium surface treatment—remains an important objective for advancing clinical applications in humans in the future.

## 5. Conclusions

The aim of this study was to assess the bone augmentation at bone defects of inbred rats by allogeneic implantation of cotton-like bone graft material seeded with DFAT cells. The following results were obtained:

1. CT images taken four weeks after implantation demonstrated a 1.25-fold increase in hard tissue formation relative to the control group.
2. In HE-stained tissue specimens, eosin-positive hard tissue formation was observed surrounding the cotton-like bone graft material, closely associated with the transplanted titanium bar.
3. Histomorphometric analysis indicated that the BIC area in the DFAT group was approximately 2.5 times greater than that of the control group.
4. The hard tissue formed adjacent to the titanium exhibited a level of bone maturity comparable to cortical bone.

These findings suggest that DFAT cells, characterized by their ease of harvest and high purity as multipotent cells, are effective for osseointegration via bone augmentation, making them a valuable cell source in the dental and oral fields. However, since applying this to humans is not currently practical, there are still challenges in improving rigorous protocols regarding the condition of the cells, transplantation methods, transplantation duration, and evaluation methods.

## Data Availability

The data presented in this study are available in the article.

## Abbreviations

The following abbreviations are used in this manuscript:

GBR	Guided bone regeneration
MSCs	Mesenchymal stem cells
DFAT	Dedifferentiated fat
ASCs	Adipose-derived stromal cells
PLGA	Poly(lactic-co-glycolic acid)
β-TCP	β-Tricalcium Phosphate
PBS	Phosphate-buffered saline
DMEM	Dulbecco’s modified Eagle’s medium
FBS	Fetal bovine serum
CT	Micro–computed tomography
BV	Bone volume
ROI	Region of interest
HE	Hematoxylin–eosin
VG	Villanueva–Goldner
BIC	Bone-to-implant contact
